# Personality, inflammatory risk, and the evolution of psychocardiological comorbidity

**DOI:** 10.1192/j.eurpsy.2026.10360

**Published:** 2026-07-31

**Authors:** V. R. Enătescu

**Affiliations:** Neuroscience, University of Medicine and Pharmacy “Victor Babes” Timisoara, Timisoara, Romania

## Abstract

**Symposium abstract:**

Over time, identifying a specific personality type that confers increased vulnerability to cardiac disease has proven challenging. Moreover, specific personality traits may also predispose individuals to major psychiatric disorders, exerting cumulative effects that increase the risk of psychosomatic conditions. In this context, coronary heart disease can be regarded as a prototypical model of these underlying psychobiological mechanisms. The concept of Type D personality was introduced to characterize enduring psychological traits observed in individuals with coronary heart disease who have a highly persistent tendency to experience negative emotions throughout life. Individuals with Type D personality exhibit heightened sensitivity of the hypothalamic–pituitary–adrenal (HPA) axis, leading to overreacting neuroendocrine responses, systemic immune activation, and chronic inflammation. This inflammatory state contributes to endothelial dysfunction and promotes the transformation of circulating immune cells into lipid-laden foam cells, thereby accelerating atherosclerotic processes within the coronary arteries. Over time, sustained neuroinflammation leads to reduced levels of monoamines and neurotrophic factors in the brain, as well as increased excitotoxicity. Altogether, these mechanisms may account for the higher prevalence of cardiac comorbidity and poorer cardiovascular outcomes observed in individuals with Type D personality compared with their non–Type D counterparts.

**Keywords:**

type D personality, inflammatory mechanisms, coronary heart disease

**References:**

1. Kupper N, Denollet J. Type D Personality as a Risk Factor in Coronary Heart Disease: a Review of Current Evidence. Curr Cardiol Rep. 2018 Sep 12;20(11):104.

2. Libby P. Inflammation and cardiovascular disease mechanisms. Am J Clin Nutr. 2006 Feb;83(2):456S-460S.

3. Simats A, Sager HB, Liesz A. Heart-brain axis in health and disease: role of innate and adaptive immunity. Cardiovasc Res. 2025 Apr 8;120(18):2325-2335.

**Disclosure of Interest:**

None Declared

